# Plantar pressure and falling risk in older individuals: a cross-sectional study

**DOI:** 10.1186/s13047-023-00612-4

**Published:** 2023-03-21

**Authors:** Yifeng Yan, Jianlin Ou, Hanxue Shi, Chenming Sun, Longbin Shen, Zhen Song, Lin Shu, Zhuoming Chen

**Affiliations:** 1 Department of Rehabilitation Medicine, The First Affiliated Hospital of Jinan University, Guangzhou, China; 2 School of Future Technology, South China University of Technology, Guangzhou, China; 3 School of Microelectronics, South China University of Technology, Guangzhou, China; 4 Institute of Modern Industrial Technology of SCUT in Zhongshan, Zhongshan, China; 5 Pazhou Lab, Guangzhou, China

**Keywords:** Fall risk, Elderly people, Plantar pressure

## Abstract

**Background:**

Falls are commonplace among elderly people. It is urgent to prevent falls. Previous studies have confirmed that there is a difference in plantar pressure between falls and non-falls in elderly people, but the relationship between fall risk and foot pressure has not been studied. In this study, the differences in dynamic plantar pressure between elderly people with high and low fall risk were preliminarily discussed, and the characteristic parameters of plantar pressure were determined.

**Methods:**

Twenty four high-fall-risk elderly individuals (HR) and 24 low-fall-risk elderly individuals (LR) were selected using the Berg Balance Scale 40 score. They wore wearable foot pressure devices to walk along a 20-m-long corridor. The peak pressure (PP), pressure time integral (PTI), pressure gradient (maximum pressure gradient (MaxPG), minimum pressure gradient (MinPG), full width at half maximum (FWHM)) and average pressure (AP) of their feet were measured for inter-group and intra-group analysis.

**Results:**

The foot pressure difference comparing the high fall risk with low fall risk groups was manifested in PP and MaxPG, concentrated in the midfoot and heel (*p* < 0.05), while the only time parameter, FWHM, was manifested in the whole foot (*p* < 0.05). The differences between the left and right foot were reflected in all parameters. The differences between the left and right foot in LR were mainly reflected in the heel (*p* < 0.05), while it in the HR was mainly reflected in the forefoot (*p* < 0.05).

**Conclusions:**

The differences comparing the high fall risk with low fall risk groups were mostly reflected in the midfoot and heel. The HR may have been more cautious when landing. In the intra-group comparison, the difference between the right and left foot of the LR was mainly reflected during heel striking, while it was mainly reflected during pedalling in the HR. The sensitivity of PP, PTI and AP was lower and the newly introduced pressure gradient could better reflect the difference in foot pressure between the two groups. The pressure gradient can be used as a new foot pressure parameter in scientific research.

## Background

The aging of the global population is becoming more and more serious, and with it the related problems [1]. Studies showed approximately 28% of elderly people aged 65 and above fall every year [2, 3] and its incidence is higher among the older group. Falls occur with high frequency, great harmfulness and many sequelae, which bring a heavy burden to society [4, 5]. And fall fatalities have increased over the past decade [6]. As such, fall prevention among elderly people and reduction of fall rate is of great urgency.

The increased risk of falls is associated with an increment of gait variability [7]. The ability to walk is one of the most natural and basic forms of human movement and a prerequisite for independent human activity and self-care. With age growing, gait changes because of the alteration of balance control, degeneration of the musculoskeletal system, and diminished sensorimotor function. As gait changes, plantar pressure will change correspondingly. Therefore, the plantar pressure in walking is often used to study normal and abnormal gait characteristics. Plantar pressure has now been applied widely in studies related to falls in elderly people. The plantar center of pressure (COP) trajectory is the most widely used. Estevez-Pedraza presents a statistical model for estimating fall risk from the COP data [8]. Also, studies by Pizzigalli concluded that certain swaying characteristics of silent posture, particularly in the medial-lateral direction, are significantly different from those of non-falling and falling people [9]. Muir's study indicated that COP displacement was significantly worse in those who fell [10]. Although a recent systematic review concluded that certain COP measures may be linked to a fall in certain circumstances rather than others [11], the force platform parameters may indeed be useful for fall risk prediction [12, 13]. However, the aforementioned studies have focused on static balance posture control and lacked studies on dynamic gait parameters, with only Mickle showing that compared to non-fallers, fallers featured significantly higher peak pressures and pressure time integrals [14]. Pol found the fallers had greater medial midfoot, medial forefoot, and bunion loading [15]. Mickle and Pol have used the "two-step method" to test plantar pressure, with equipment limited to the laboratory.

In addition, we found that previous studies have focused on falls between fallers and non-fallers when a primary injury from a fall has already developed. Whereas the most important thing against falls is the prevention of falls, there are few studies on the risk and foot pressure before falls. Plantar pressure is an essential feature during walking, and it has a good potential to improve our awareness before a fall, and future wearable wireless plantar pressure devices will be more convenient [16]. Therefore, based on the wearable intelligent footwear system [17–20], a preliminary study was made on plantar pressure in elderly people with a high or low risk of falling.

The two aims of this study were to investigate whether there are differences in dynamic plantar pressure among elderly people at high and low risk of falls; if there are differences, we tried to search for the plantar pressure characteristic parameters.

## Methods

A cross-sectional study was applied.

### Participants

Participants were recruited from January 2021 to May 2022 at the Department of Rehabilitation Medicine of the First Affiliated Hospital of Jinan University, Guangzhou, China. Participants were primarily outpatient follow-up patients and hospital attendants. Recruitment announcements were posted on the department bulletin boards and outpatient department. Participants of interest were screened according to inclusion and exclusion criteria, and those who met the requirements were invited to participate in the study.

Inclusion criteria: (a) age 65 or older; (b) capable of independent walking for 3 min without assistance; (c) clear consciousness, able to cooperate with the assessment, Mini-mental State Examination score > 24; (d) with available informed consent. Exclusion criteria: (a) Those with foot injuries, deformities and other conditions that temporarily affect their daily walking; (b) People receiving trunk or lower limb therapy that affect lower extremity biomechanics; (c) patients with serious or unstable cardiac, pulmonary, renal and other medical diseases who can not tolerate the study; (d) patients with a history of mania, delirium and other psychiatric disorders who cannot cooperate to complete the test.

According to previous study [15] and pre-experiment results, it is expected that the combined sample standard deviation σ is 1.36, and the difference between the two groups' means δ is 1.1. Bilateral α = 0.05 is set, and power (1-β) is 80%. The sample size was calculated according to the formula (1), and n = 24 were obtained. So each group requires 24 samples.

1 $$n=\frac{2{\left({z}_{\alpha }+{z}_{\beta }\right)}^{2}*{\sigma }^{2}}{{\delta }^{2}}$$

Finally, forty-eight elderly people were selected for general data collection and Berg Balance Scale (BBS) assessment, of whom 24 BBS scored ≤ 40 for the high fall risk group (HR) and the other 24 BBS scored > 40 for the low fall risk group (LR). The general data includes sex, age, height, body weight and body mass index.

### Apparatus and equipment

**Fig. 1:**
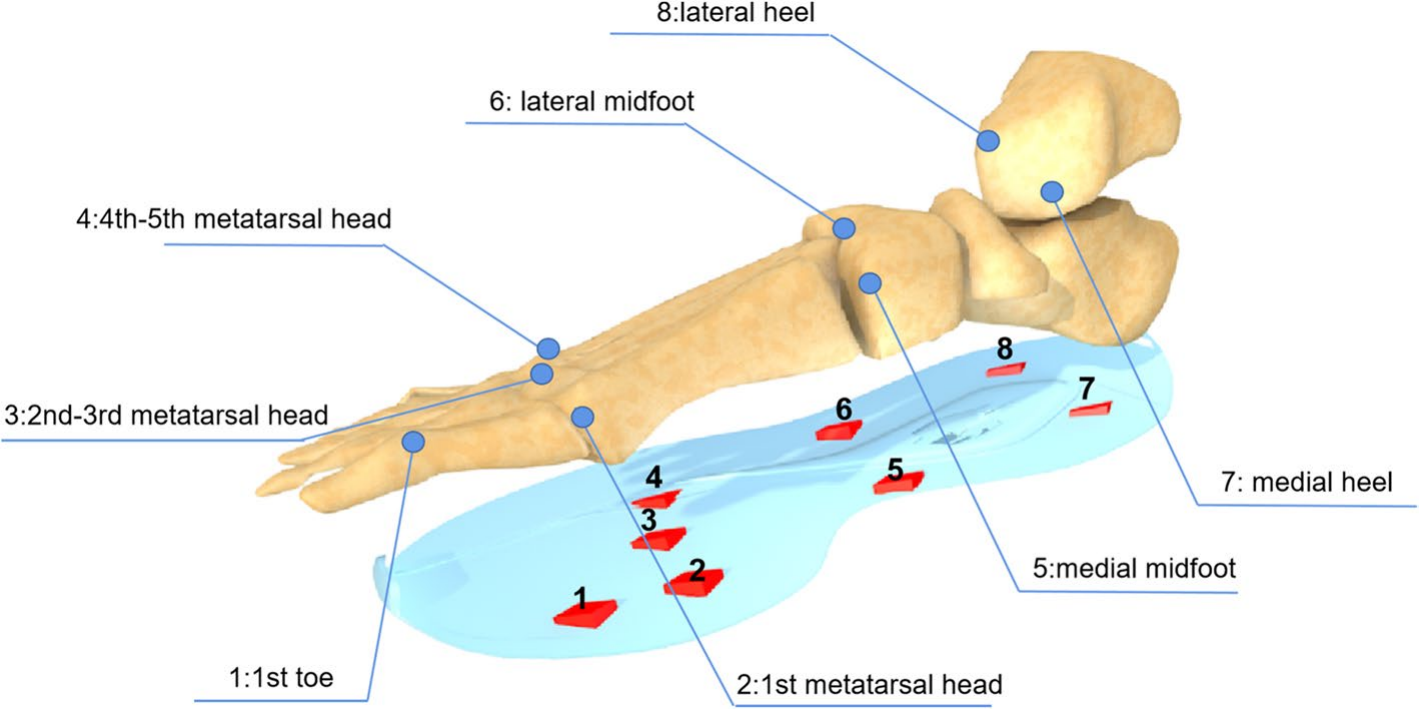
Position of eight pressure sensors. The right foot is shown as an example

**Fig. 2:**
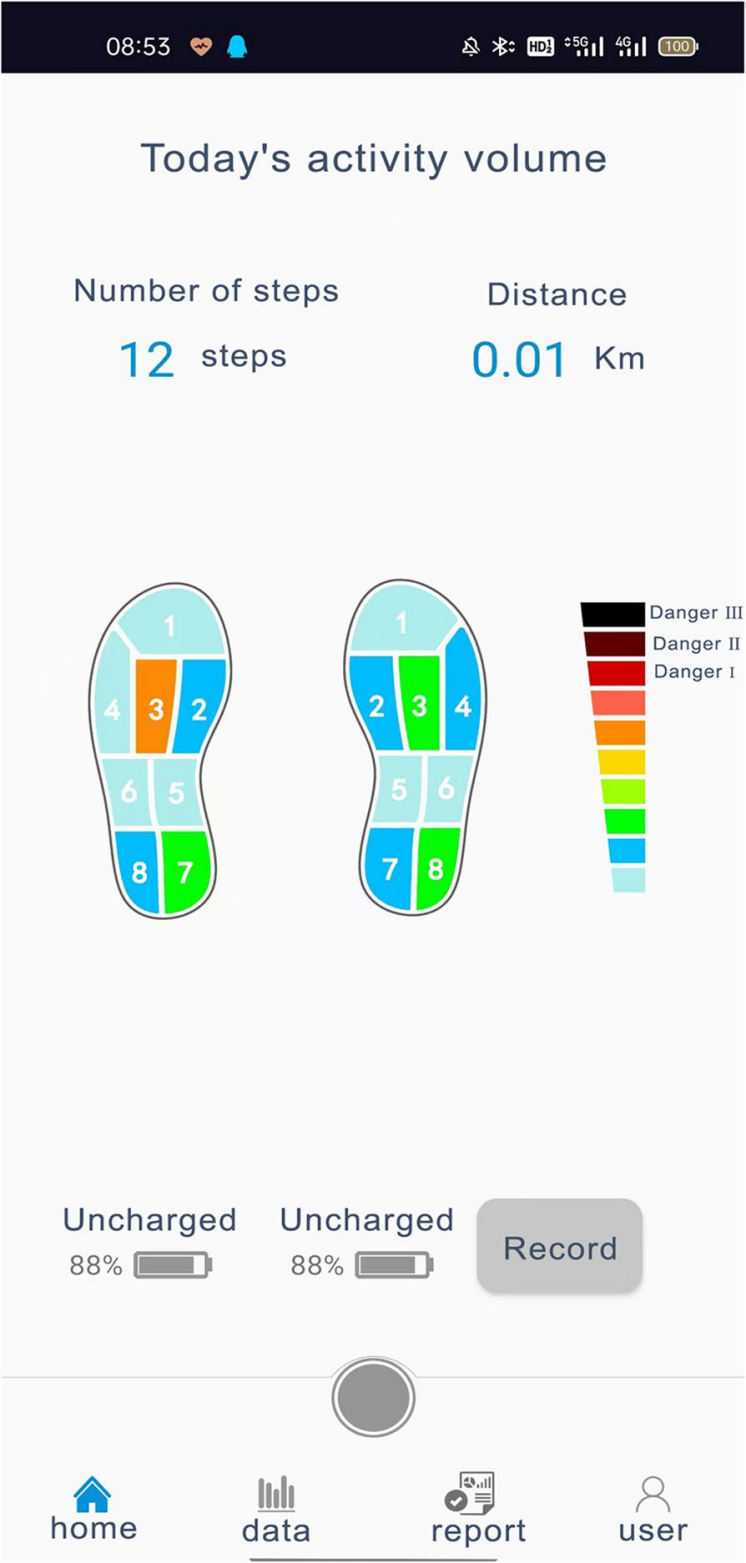
Different colours according to the pressure grading. During the test, the color will change dynamically with the pressure

The plantar pressure was detected using a wearable intelligent footwear system developed in cooperation with the Human Data Science Engineering Center of South China University of Technology and Zhongshan Super Sense Technology Co [19, 20]. As shown in Fig. 1, there are eight pressure sensors at different points in the insole of each foot. The pressure sensor has the characteristics of a short response time, large range, high sensitivity, and high wear resistance [17]. Wirelessly connected mobile phones can receive real-time datums collected by the footwear system. And the phone APP sets different colours according to the pressure grading, indicating the dynamic changes of plantar pressure, as shown in Fig. 2. It was confirmed that satisfactory accuracy, repeatability and wearing comfort was showed by this intelligent footwear system [18].

### Procedure

During the experiment, the researcher provided uniform cotton socks and the participants were asked to choose the right size of socks and intelligent shoes to ensure that their feet would not slide in the shoes while walking. Before the experiment began, the participants wore cotton socks and intelligent shoes for 1-2 min to adapt. During the formal experiment, participants need to walk for more than two minutes along a 20-m corridor at their normal gait and usual walking speed. The experiments were supervised by one investigator, with no physical contact or verbal induction. Each experiment was supervised by the same investigator. And participants went through the whole process in one day.

### Observation criteria and data extraction

**Fig. 3:**
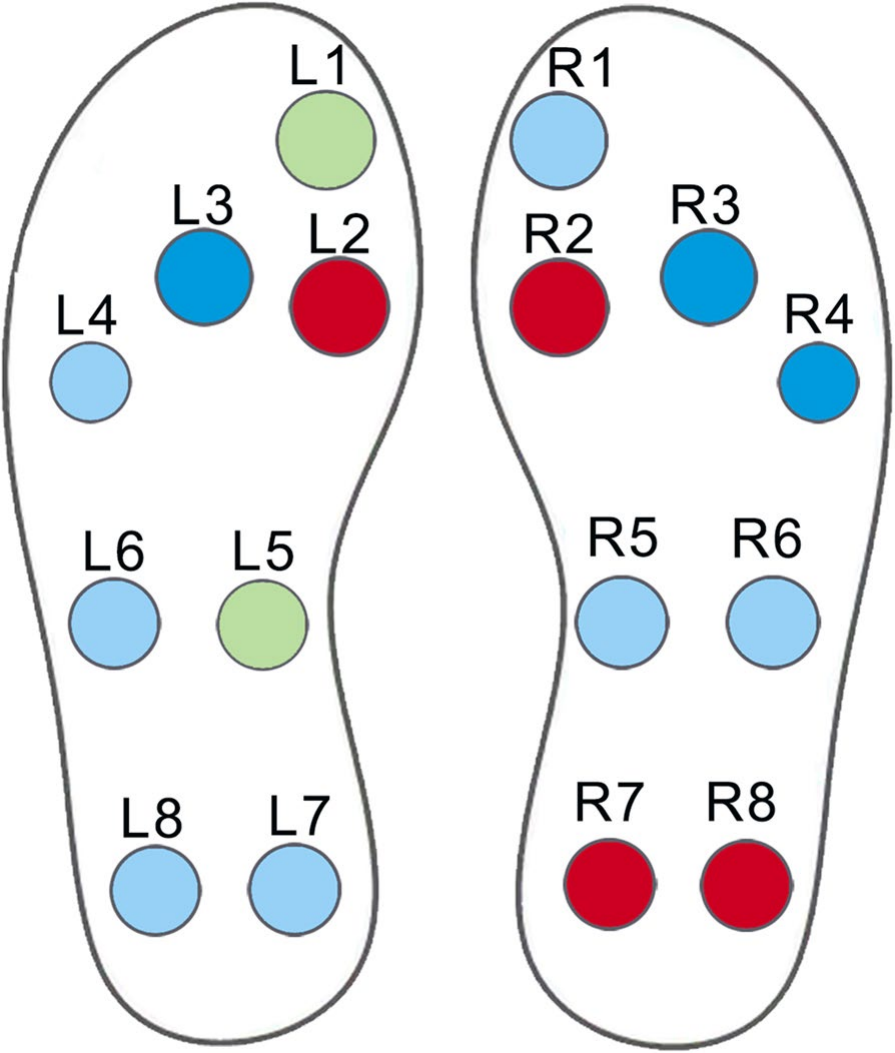
Eight regions for analysis. These 8 regions correspond to the position of the pressure sensor one by one

Based on previous studies combined with plantar mechanics, the plantar area was divided into 8 regions for analysis: 1st toe (T1), 1st metatarsal head (M1), 2nd-3rd metatarsal head (M2-3), 4th-5th metatarsal head (M4-5), medial midfoot (MMF), lateral midfoot (LMF), medial heel (MH), and lateral heel (LH), as shown in Fig. 3.

The raw plantar pressure data is exported from the smart terminal mobile APP background. After weight normalization and identification of valid gait cycles, for each foot region, we calculate the following parameters: peak pressure (PP), pressure-time integral (PTI), pressure gradient (maximum pressure gradient (MaxPG), minimum pressure gradient (MinPG), full width at half maximum (FWHM)), and average pressure (AP). In this study, we used the characteristic calculation method of Botros [21] and Dongran Wang [17] et al.

For each plantar region i, where i = 1, 2,…, 16 and i ∈  [1-8] for the left foot and i ∈  [9-16] for the right foot, Pi(m) is the pressure datums for each sample suiting to the region, where m = 1,2,…, M. M is the length of the sample data for one valid gait cycle. Then for each Pi(m), calculate 12 feature F_ri_ and perform the average of individual effective gait cycles, where r = 1, 2,…, 12.

Taking the left foot as an example, with r ∈  [1-6], the 1st parameter PP can be calculated by Eq. (2).

2 $${\mathrm{F}}_{1\mathrm{i}}{= }_{\mathrm{m}\in \left[1,\mathrm{M}\right]}^{\mathrm{ max}}{\mathrm{P}}_{\mathrm{i}}\left(\mathrm{m}\right){|}_{\mathrm{L}}$$

The 2nd parameter PTI can be calculated by Eqs. (3).

3 $${\mathrm{F}}_{2\mathrm{i}}= {\sum }_{\mathrm{m}=1}^{\mathrm{M}-1}{(\mathrm{P}}_{\mathrm{i}}\left(\mathrm{m}\right){|}_{\mathrm{L}}+{\mathrm{P}}_{\mathrm{i}}(\mathrm{m}+1){|}_{\mathrm{L}}).\Delta \mathrm{m}/2$$

The 3rd parameter MaxPG and the 4th parameter MinPG are calculated by Eqs. (4) and (5), respectively.

4 $${\mathrm{F}}_{3\mathrm{i}}{= }_{\mathrm{m}\in \left[1,\mathrm{M}\right]}^{\mathrm{ max}}{\nabla \mathrm{P}}_{\mathrm{i}}\left(\mathrm{m}\right){|}_{\mathrm{L}}{= }_{\mathrm{m}\in \left[1,\mathrm{M}\right]}^{\mathrm{ max}}{ [\mathrm{ P}}_{\mathrm{i}}\left(\mathrm{m}\right){|}_{\mathrm{L }}{ -\mathrm{ P}}_{\mathrm{i}}\left(\mathrm{m}-1\right){|}_{\mathrm{L}}]/\Delta \mathrm{m}$$

5 $${\mathrm{F}}_{4\mathrm{i}}{= }_{\mathrm{m}\in \left[1,\mathrm{M}\right]}^{\mathrm{ min}}{\nabla \mathrm{P}}_{\mathrm{i}}(\mathrm{m}){|}_{\mathrm{L}}{= }_{\mathrm{m}\in \left[1,\mathrm{M}\right]}^{\mathrm{ min}}{ [\mathrm{ P}}_{\mathrm{i}}\left(\mathrm{m}\right){|}_{\mathrm{L }}{ -\mathrm{ P}}_{\mathrm{i}}(\mathrm{m}-1){|}_{\mathrm{L}}]/\Delta \mathrm{m}$$

The 5th parameter FWHM can be calculated by Eqs. (6) and (7), where m_i1_ and m_i2_ denote the longness of the sample datums when the pressure value is half of the PP.

6 $${\mathrm{F}}_{5\mathrm{i}}={\mathrm{m}}_{\mathrm{i}2}{|}_{\mathrm{L}}{ -\mathrm{ m}}_{\mathrm{i}1}{|}_{\mathrm{L}}$$

7 $${\mathrm{P}}_{\mathrm{i}}{(\mathrm{m}}_{\mathrm{i}2}{)|}_{\mathrm{L}}={\mathrm{P}}_{\mathrm{i}}{(\mathrm{m}}_{\mathrm{i}1}{)|}_{\mathrm{L}}=0.5\times {\mathrm{F}}_{1\mathrm{i}}$$

The sixth parameter AP can be calculated by Eq. (8).

8 $$\overline{{\mathrm{F} }_{6\mathrm{i}}}=\frac{1}{\mathrm{m}}\sum_{\mathrm{r}=1}^{\mathrm{m}}{\mathrm{P}}_{\mathrm{i}}\left(\mathrm{m}\right){|}_{\mathrm{L}}$$

### Statistical analysis

Except for parameter calculations, all data were processed by IBM SPSS Statistics for Windows, Version 27.0 (IBM Corp, Armonk, NY, USA). Data were expressed as mean ± SD or median (Q1, Q3), as appropriate. Also, group comparison was made using two independent sample t-test or Mann-Whitney U test. Besides, paired-sample t-test and Wilcoxon test were made for within-group comparisons. The significance level was set at *α* = 0.05.

### Ethical considerations

Approval was granted by the Medical Ethics Committee of the First Affiliated Hospital of Jinan University on December 23, 2020 (KY-2020-087). All participants were informed of the study purpose, procedure, anonymity and confidentiality of participation, and received written informed consent.

## Results

### Baseline data

The baseline data, including sex, age, height, body weight and body mass index of the two groups were compared, and the differences were not statistically significant (*p* > 0.05). As shown in Table 1.

**Table 1:** Comparison of baseline information of high and low fall risk groups

Group	Number of cases	Sex (cases)		Age	Height	Body weight	Body mass index
		men	women	(age, $\overset-{\mathrm x}$ ± s)	(cm, $\overset{-}{\mathrm x}$ ± s)	(kg, $\overset-{\mathrm x}$ ± s)	(kg/m^2^, $\overset-{\mathrm x}$ ± s)
LR	24	10	14	72.63 ± 5.97	159.88 ± 7.77	60.17 ± 8.43	23.55 ± 2.91
HR	24	15	9	76.33 ± 6.96	163.38 ± 8.68	62.46 ± 8.50	23.41 ± 2.88
*P* value		0.149		0.054	0.148	0.353	0.875

*LR* Low fall risk group, *HR* High fall risk group

### Peak pressure

**Fig. 4:**
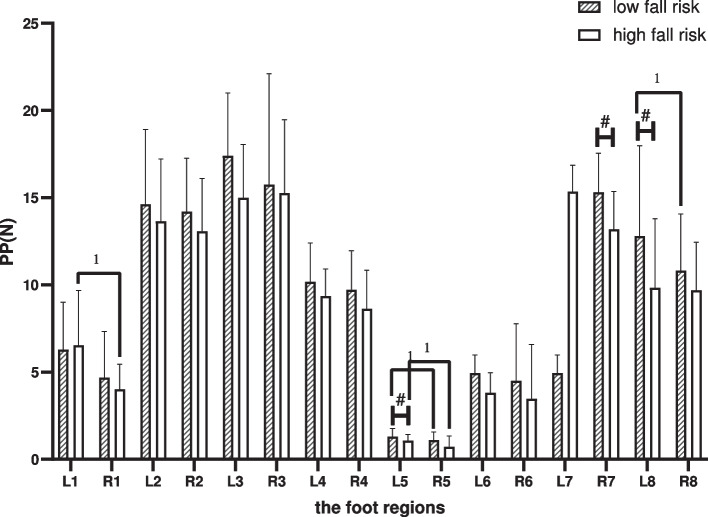
Between-group and within-group effects of PP in elderly people with high and low fall risk. Plot by the median (Q1,Q3). ^1^where significant at *p* < .05 for pared-samples T-test or Wilcoxon paired test. ^#^where significant at *p* < .05 for independent-samples T-test or Mann-Whitney U test

Between-group comparison: Compared with the LR, the PP was reduced in the left-MMF, left-LH and right-MH in the HR (*p* < 0.05). Within-group comparison: In the LR, differences comparing the left foot with the right foot were shown in the LH and MMF (*p* < 0.05). In the HR, it was shown in the MMF and the T1 (*p* < 0.05), as shown in Fig. 4.

### Pressure–time integral

**Fig. 5:**
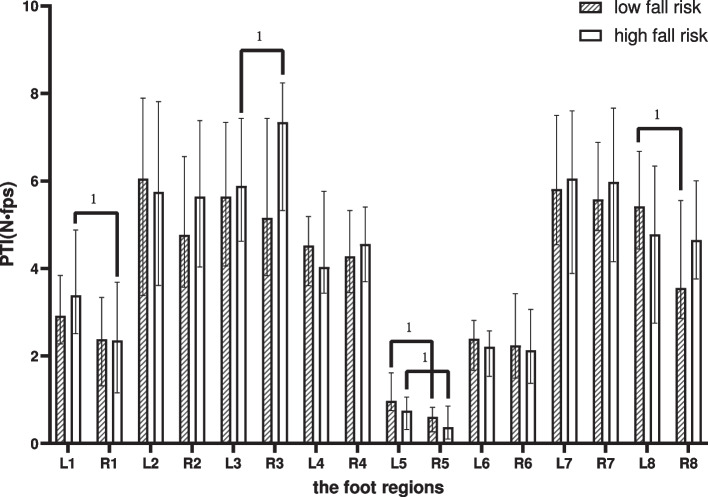
Between-group and within-group effects of PTI in elderly people with high and low fall risk. Plot by the median (Q1,Q3). ^1^where significant at *p* < .05 for pared-samples T-test or Wilcoxon paired test

Within-group comparison: In the LR, differences comparing the left foot with the right foot were shown in the LH and MMF (*p* < 0.05). In the HR, it was shown in the T1, the M2-3 and the MMF (*p* < 0.05) (Fig. 5). Comparison between groups: none of them was statistically different.

### Full width at half maximum

Comparison between groups: Compared with the LR, the FWHM increased in elderly people in the HR in the following regions (*p* < 0.05): the left-T1, the left-M1, the left-M2-3, the left-M4-5, the left-LMF, the right-M1, the right-M2-3, the right-M4-5, the right-LMF, the right-MH, the right-LH.

**Fig. 6:**
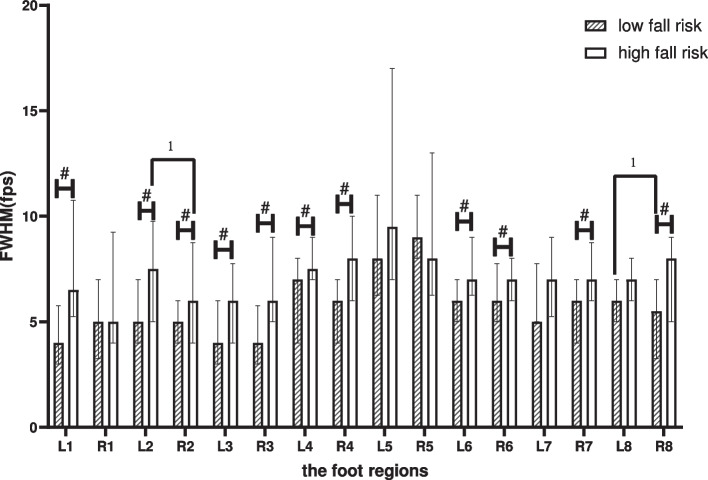
Between-group and within-group effects of FWHM in elderly people with high and low fall risk. Plot by the median (Q1,Q3). ^1^where significant at *p* < .05 for pared-samples T-test or Wilcoxon paired test. ^#^where significant at *p* < .05 for independent-samples T-test or Mann-Whitney U test

Within-group comparison: in the LR, the difference comparing the left foot with the right foot was shown on the LH (*p* < 0.05). In the HR, it was shown at the M1 (*p* < 0.05), as shown in Fig. 6.

### Maximum pressure gradient

Between-group comparison: Compared with the LR, the MaxPG in elderly people in the HR decreased in the following regions (*p* < 0.05): left-LMF, left-MH, left-LH, right-LMF, right-MH, and right-LH.

**Fig. 7:**
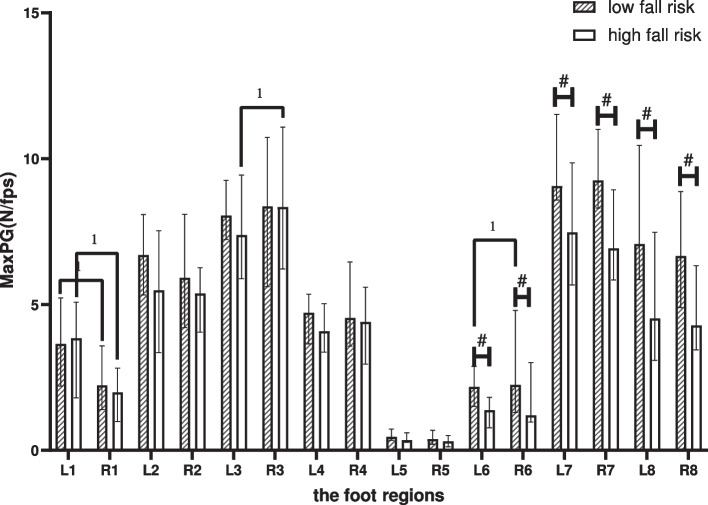
Between-group and within-group effects of MaxPG in elderly people with high and low fall risk. Plot by the median (Q1,Q3). ^1^where significant at *p* < .05 for pared-samples T-test or Wilcoxon paired test. ^#^where significant at *p* < .05 for independent-samples T-test or Mann-Whitney U test

Within-group comparison: In the LR, differences comparing the left foot with the right foot were shown in the T1 and LMF (*p* < 0.05). In the HR, it was shown in the T1 and the M2-3 (*p* < 0.05), as shown in Fig. 7.

### Minimum pressure gradient

Within-group comparison: only in the LR, the left and right feet of the M4-5 showed differences (*p* < 0.05). None of the between-group comparisons was statistically different.

### Average pressure

**Fig. 8:**
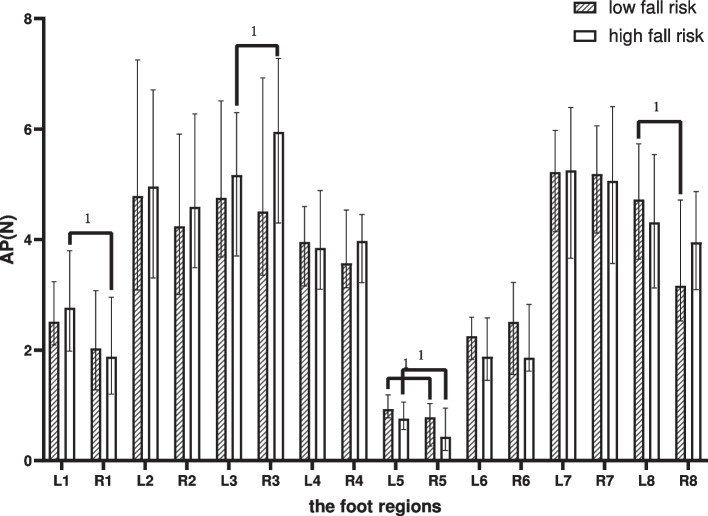
Between-group and within-group effects of AP in elderly people with high and low fall risk. Plot by the median (Q1,Q3). ^1^where significant at *p* < .05 for pared-samples T-test or Wilcoxon paired test

Within-group comparison: in the LR, differences comparing the left foot with the right foot were shown in the LH and MMF (*p* < 0.05). As shown in Fig. 8, in the HR, it was shown in the T1, M2-3 and MMF (*p* < 0.05). The difference between groups was not statistical.

## Discussion

In this paper, pressure gradients are first introduced for foot pressure analysis, and it is clear from our results that PP, PTI and AP are less sensitive in high and low fall risk studies, while the spatial variation in pressure expressed by pressure gradients can rather better reflect the difference in plantar pressure on intergroup comparison. This paper prospectively investigates the relationship between fall risk and plantar pressure. The results showed that the differences between group comparison were mainly focused on midfoot and heel, so we assume that HR may have been more cautious when landing. As for the comparison within the group, the differences between the left and right foot in LR occurred mainly when the foot heel contacted the ground, while it in HR occurred mainly when the foot stroked the ground. In the within-group comparisons between the two groups, the differences in foot pressure were in different areas.

Walking is a fundamental function of human activity, and the walking ability reflects the physiological, structural, and functional state of the lower limbs and even the whole body. Plantar pressure refers to the amount of pressure exerted on the sole during standing or walking. It can be used to reflect the walking condition. Currently, the technique of foot pressure measurement has found wide application in clinical evaluation and scientific experiment, among which the most commonly used is the plantar pressure measurement platform, but the plantar pressure measurement platform is limited to the laboratory, which has a high demand for the measurement environment. In this study, we adopted a wearable intelligent footwear system developed by a research group in cooperation, which was designed with a footwear system that can be monitored in real-time during daily life, which is convenient, applicable to various living environments, conducive to further promotion and utilization and does not bring extra burden to elderly people.

### Peak pressure and pressure–time integral

The PP is one of the most commonly used variables to indicate plantar load, representing the maximum pressure value during the contact phase. In our study, the PP was smaller in the HR compared to the LR. One study showed that the PP was smaller in elderly people with falls [22]. It may be due to the slower walking velocity of elderly people [23–26] and possibly their activation of physical protection mechanisms. The differences between the two groups were concentrated on the heel both the medial and lateral, most likely because when elderly people contact the ground with the heel close to the body’s gravity center, the foot tends to be vertical [24, 27–29], thus reducing the impact force on the foot when landing [29]. These effects may be more pronounced in HR.

The PTI is also a variable frequently used to assess plantar load. It represents the accumulated effect of plantar pressure over time and can be simply interpreted as the product of pressure and contact time, which reflects the total plantar load contact value during the walking cycle. In our study, no significant differences were reflected between HR and LR, probably because PTI is a measure of the area size of the foot pressure curve over a walking period. To eliminate the influence of individual unnatural data, we adopted the calculation method of taking the average of multiple gait cycles, which may also filter out some extremely minimal differences. Even if the effective sample size is greater than 20 gait cycles, it may not accurately reflect the differences between the two groups [30]. This may also be the reason why the AP did not differ significantly between the HR and LR. Some studies have pointed out that there is a difference in the PTI between fallers and non-fallers [14, 15], but in our study no difference was shown in the history of falls between the two groups, suggesting that the difference comparing the HR with the LR may be smaller than the difference between falls and non-falls. High fall risk is not equivalent to fall history, which is the reason why we introduced a new foot pressure parameter, pressure gradient.

### Pressure gradient

The pressure gradient is a new index introduced in this study for foot pressure studies in elderly people, which was previously widely used in clinical studies of plantar pressure [31, 32]. Mueller pioneered the concept of pressure gradient, which suggests that higher pressure gradients, namely greater pressure changes in adjacent areas of the foot surface, are more damaging to plantar soft tissues [33]. Pressure gradients can be useful indicators of skin vulnus because spacial variations in high plantar pressure can recognize high concentrations of stress within the soft tissues. Theoretically, the authors believe that the introduced pressure gradient parameter is more specific and detailed, and better reflects the subtle differences comparing the HR with the LR. Our study calculated three parameters related to pressure gradients: MaxPG, MinPG and FWHM [21].

There was a difference in the MaxPG with the HR being significantly lower than the LR. This contradicts the conventional understanding that HR has a greater chance of soft tissue damage and a larger MaxPG. However, our results showed that the MaxPG was smaller in the HR, while the FWHM was found to be significantly higher. Because the FWHM refers to the difference between the two-time points at which half of the PP is reached, it indicates the time it takes for the participants to rise from the half-peak pressure to the PP and then to fall to the half-peak pressure. The significant increase in the HR indicates that the HR walking process is slower and the entire process is kept for a longer time, while the MaxPG of the HR is significantly lower than that of the LR, which further proves that elderly people with high fall risk will be more cautious during the walking process and their exposure to spatial variation of pressure will be smaller. This may also suggest that elderly people at high risk of falls do not necessarily have a higher likelihood of plantar soft tissue injury, which can be studied further and deeper later.

### Within-group comparison

As for the comparison of left and right feet in each index group, it can be found regardless of the high or low fall risk group, there was a difference between their left and right feet, and the asymmetry of both feet led to a decrease in their balance ability, which is in line with previous studies [24, 34]. Our study found that the differences comparing the left foot with the right foot in the LR were concentrated at the midfoot and heel, while those in the HR were concentrated at the anterior metatarsal head. It can be simply interpreted that the difference between the feet in the LR occurred when the foot contacted the ground with the heel, and it in the HR occurred when the foot pedalled. This may be related to changes in the toe grip force of the dominant foot in the older [35]. It also may be related to the enhanced toe flexion of the long and short toe flexors. Their enhanced toe flexion could compensate for reduced plantar fascia function in the 2nd and 3rd metatarsals, enhance forefoot stirrups, and increase proprioceptive feedback to the plantar side of the foot, improving postural stability in elderly people [36]. We also have the right to assume that the change in the walking style of elderly people occurs during the heel landing in the early stages of fall risk, and that the forefoot pedalling style changes when the fall risk progresses to a certain level.

### Between-group comparison

The difference in foot pressure between HR and LR is mainly the reduction of HR reflected in the midfoot and heel by PP and MaxPG (FWHM belongs to a time parameter), which is different from similar studies conducted by previous researchers. Mickle believed that the PP of non-fallers was significantly lower than that of fallers [14], but it is aimed at the total plantar pressure value and is related to foot pain, In our study, all the subjects had no foot pain, and there was no difference in the fall history between our two groups of subjects. Secondly, we needed to wear socks and shoes during the experiment, while Mickle's experiment was barefoot walking. And walking barefoot exhibited different plantar pressure than walking in shoes [25]. Whereas Pol found that fallers had higher PTI in the medial foot compared to non-fallers [15]. Brenton-Rule also found higher plantar pressures in fallers among adults with rheumatoid arthritis [37]. A recent study also showed that an increase in PTI is associated with falling fears [38]. However, previous studies focused on dividing experimental groups based on fall history as fall risk, and most of the experimental methods adopted the "one-step method" or "two-step method" on the force measurement platform, which is different from the experimental design of this study. In this study, the fall risk scale was adopted, and the walking state in daily life was restored as much as possible in the experiment, which is also the innovation of this study.

## Limitation

This study also has certain limitations. Firstly, when including subjects, we simply excluded subjects with obvious foot diseases that would affect daily walking, without considering the subjects' foot structure in detail, such as flat feet, etc., which may affect the results of plantar pressure. Secondly, our limited sample sizes fail to ensure a fully representative and widespread conclusion. Yet, it’s underpinned by medical theoretical knowledge, which could offer some clues to the research. Thirdly, Plantar pressure was not standardized by shoe size. Although most of our subjects adopted shoe size 37 for women and 41 for men, and our analysis was a region-specific analysis, our research group believes that the relationship between shoe size and foot pressure needs further investigation. This may also be another research direction for us in the future.

## Conclusion

These results preliminary suggest that there were indeed differences in plantar pressure between high and low fall risk in older adults and that plantar pressure may be used to determine fall risk, especially pressure gradient. In the subsequent fall prevention studies, we believe that focusing on the prior study, that is, starting from the fall risk, rather than distinguishing whether a people falls from the history of falls can fundamentally solve a series of problems caused by falls in elderly people. Meanwhile, the successful use of pressure gradient also prompts us to pay attention to the analysis and application of parameters reflecting small changes in plantar pressure, especially in real studies with small differences.

## Data Availability

The datasets analyzed during the current study are available from the corresponding author upon reasonable request.

## Abbreviations

COP: Plantar center of pressure
BBS: Berg Balance Scale
HR: High fall risk group
LR: Low fall risk group
T1: 1St toe
M1: 1St metatarsal head
M2-3: 2Nd-3rd metatarsal head
M4-5: 4Th-5th metatarsal head
MMF: Medial midfoot
LMF: Lateral midfoot
MH: Medial heel
LH: Lateral heel
PP: Peak pressure
PTI: Pressure-time integral
MaxPG: Maximum pressure gradient
MinPG: Minimum pressure gradient
FWHM: Full width at half maximum
AP: Average pressure
